# Brain Dynamics of Aging: Multiscale Variability of EEG Signals at Rest and during an Auditory Oddball Task^1^,^2^,^3^

**DOI:** 10.1523/ENEURO.0067-14.2015

**Published:** 2015-06-03

**Authors:** Rita Sleimen-Malkoun, Dionysios Perdikis, Viktor Müller, Jean-Luc Blanc, Raoul Huys, Jean-Jacques Temprado, Viktor K. Jirsa

**Affiliations:** 1 Aix-Marseille Université, Inserm, Institut de Neurosciences des Systèmes UMR_S 1106, 13385, Marseille, France; 2 Aix-Marseille Université, CNRS, Institut des Sciences du Mouvement UMR 7287, 13288, Marseille, France; 3 Max Planck Institute for Human Development, Center for Lifespan Psychology, 14195, Berlin, Germany; 4 CNRS, 13402, Marseille, France

**Keywords:** aging, brain dynamics, brain signal variability, complexity, EEG, multiscale entropy

## Abstract

The present work focused on the study of fluctuations of cortical activity across time scales in young and older healthy adults. The main objective was to offer a comprehensive characterization of the changes of brain (cortical) signal variability during aging, and to make the link with known underlying structural, neurophysiological, and functional modifications, as well as aging theories. We analyzed electroencephalogram (EEG) data of young and elderly adults, which were collected at resting state and during an auditory oddball task. We used a wide battery of metrics that typically are separately applied in the literature, and we compared them with more specific ones that address their limits. Our procedure aimed to overcome some of the methodological limitations of earlier studies and verify whether previous findings can be reproduced and extended to different experimental conditions. In both rest and task conditions, our results mainly revealed that EEG signals presented systematic age-related changes that were time-scale-dependent with regard to the structure of fluctuations (complexity) but not with regard to their magnitude. Namely, compared with young adults, the cortical fluctuations of the elderly were more complex at shorter time scales, but less complex at longer scales, although always showing a lower variance. Additionally, the elderly showed signs of spatial, as well as between, experimental conditions dedifferentiation. By integrating these so far isolated findings across time scales, metrics, and conditions, the present study offers an overview of age-related changes in the fluctuation electrocortical activity while making the link with underlying brain dynamics.

## Abstract (precis)

Recently, the study of brain signal fluctuations is widely put forward as a promising entry point to characterize brain dynamics in health and disease. Although interesting results have been reported regarding how variability of brain activations can serve as an indicator of performance and adaptability in elderly, many uncertainties and controversies remain with regard to the comparability, reproducibility, and generality of the described findings, as well as the ensuing interpretations.

## Significance Statement

Recently, the study of brain signal fluctuations is widely put forward as a promising entry point to characterize brain dynamics in health and disease. Although interesting results have been reported regarding how variability of brain activations can serve as an indicator of performance and adaptability in elderly, many uncertainties and controversies remain with regard to the comparability, reproducibility, and generality of the described findings, as well as the ensuing interpretations. Following a systematic investigation of these issues by using a large set of metrics and different experimental conditions, our results draw an overview of age-related changes of the magnitude and structure of brain fluctuations, which integrate well with known structural and functional alterations, as well as the main aging theories.

## Introduction

The view that variability in brain activity serves a functional role is gaining increasing support (Ghosh et al., 2008; Deco et al., 2009, 2011, 2013; Garrett et al., 2011; Hong and Rebec, 2012). The characteristics of brain signal fluctuations are considered to capture the underlying complex interactions between neuronal structures and ensembles.

At rest, the brain displays a complex though spatiotemporally structured dynamics, where brain states known as resting-state networks are intermittently activated. These states are considered to be functionally meaningful because several of them have been known from task paradigms (Deco et al., 2013). As underlying mechanisms, within deterministic frameworks, heteroclinic cycles have been proposed to generate sequential transitions from one unstable equilibrium point (saddle) to another. Other deterministic approaches soften the requirement of unstable states and require linked attractive subspaces (Huys et al., 2014). These approaches are subject to noise, which seems to be pervasive at different levels of the CNS (Faisal et al., 2008). However, they do not necessarily require the latter as a generative element as do those considering that the continually fluctuating background activity, random or not, drives the multistable system through a cascade of epochs of invariant, but distinct, coordinated network activities (Hansen et al., 2015). McIntosh et al. (2010) argued that noise is linked to an increased number of functional network configurations that can be occupied in stochastic systems. This suggests that maturational changes in brain noise represent an enhancement of the functional network potential, the brain’s dynamic repertoire (Ghosh et al., 2008). Conversely, the natural process of aging, as well as disease, has been associated with an evolution toward poorer dynamics, more local interactions, and more regular fluctuations in brain and behavior (for review, see Garrett et al., 2013b; Sleimen-Malkoun et al., 2014).

In the ergodic theory framework, entropy has been theoretically demonstrated to be an nonredundant measure of dynamical systems (Adler and Weiss, 1967; Ornstein and Weiss, 1991). In empirical data, neurobehavioral variability is characterized through the magnitude (variance-derived measures) and the time structure (long-range correlations and entropy-derived metrics; Bravi, et al., 2011) of fluctuations. The main operational principle is that the healthy system exhibits complex fluctuations somewhere at a sweet spot between randomness and regularity. Such resonance-like phenomena are known as stochastic resonance and have been observed in biological systems including brain networks (Gammaitoni et al., 1998; Deco et al., 2009; McDonnell and Abbott, 2009; McDonnell and Ward, 2011). Nevertheless, most of the widely used measures cannot distinguish between deterministic and stochastic components of the dynamics. Entropy measures, for instance, are relevant for comparisons between different conditions (e.g., resting vs task) or systems (e.g., young vs old), assuming conventionally that more entropy corresponds to more complexity (Feldman and Crutchfield, 1998). Sensu stricto, this latter assumption is not always correct, at least not with single-scale measures (Costa et al., 2002, 2005).

In fMRI studies, variance-based measures (Grady and Garrett, 2014), as well as entropy measures (Liu et al., 2013; Sokunbi, 2014), have been shown to be relevant to characterize and understand the dynamics of the aging brain. In this context, multiscale analyses have also been used (Yang et al., 2013; Smith et al., 2014), although, their contribution is restricted due to the limited range of functionally meaningful scales that can be covered. Such measures are of more interest in signals with higher time resolution, as electroencephalogram (EEG) and MEG recordings, where time-scale dependence of aging effects can be revealed (McIntosh et al., 2014). Nevertheless, notwithstanding a number of converging findings showing that aging does affect the variability of brain activity, no final conclusions can be made yet concerning the nature of such changes or their link with functional and adaptive capabilities. The present study makes a helpful step in this direction by offering a consistent and coherent characterization of EEG signals in young and older adults through a multiplicity of metrics applied to both resting and task conditions. Specifically, it investigates the following: (1) the type of information that can (or cannot) be captured by the (univariate) metrics that are conventionally used to characterize brain signals, (2) the distinction between multiscale changes in the magnitude of fluctuations and their structure in time, (3) the correspondences between different classes of metrics with regard to age-related modifications in brain activity, (4) the comparability between aging effects on resting and task-evoked brain fluctuations, and (5) the extent to which changes in brain fluctuations can be linked to structural and functional changes occurring in the aging brain.

## Methods

### Participants

Participants were recruited through announcements at schools in Saarland and at the Saarland University. They received a compensation of 7.5 Euro per hour. All the participants were right-handed, had no reported history of head or neurological disorders, and none were on medication. The studied sample consisted of 31 young (Y; mean age = 22.7, SD = 1.6, age range = 18.8–25.1 years, 14 females), and 28 old adults (O; mean age = 67.8, SD = 3.0, age range = 63.9–74.5 years, 14 females). Participants of all ages were able to sustain their attention for the entire duration of the experiment, and they all underwent a psychological and audiological assessment prior to their enrollment. The used protocol was in accordance with the regulation of the local ethics committee. All participants volunteered for this experiment and gave their written informed consent prior to their inclusion in the study.

### Procedure

The EEG measurement began with a 3 min resting-state recording (1.5 min with eyes closed, and 1.5 min with eyes open) and was followed by the auditory oddball task. During the task, participants were seated comfortably on a chair in an electrically shielded room, with their eyes closed. They heard two different tone beeps: a frequent 1000 Hz tone as a standard stimulus and a rare 800 Hz tone as a deviant stimulus. The standard and deviant stimuli were presented binaurally (with a probability of 0.8 and 0.2 for standard and deviant, respectively) through headphones (Sony DJMDR-V300) at 70 dB SPL with duration of 70 ms (including 10 ms rise and fall time). Stimuli were generated with the software Audacity 1.2.4. The interstimulus interval ranged from 1200 to 1500 ms. There were two different experimental conditions: passive listening (unattended) and active counting (attended). In the first condition, participants merely listened to the tone beeps without any response, whereas in the second condition, they had to attend to stimuli and to count the deviant tones. After the session, they were asked to report their counting results. Each experimental condition contained 152 standard tones and 38 deviant tones presented in a pseudorandom order fixed for all participants. The order of the conditions was always the same, with the active counting condition following the passive listening condition. For this study, we considered three conditions, all with eyes closed: resting state (R), auditory oddball task without counting (OnC), and auditory oddball with counting (OC). The condition of resting state with eyes open was not included because it differed largely in its frequency content compared to all other conditions, which interfered with tasks contrasts. Instead, we focused on studying differences under comparable conditions along the axis of increasing attentional and task demands.

### EEG recordings and preprocessing

The electroencephalogram (EEG) was recorded from 58 Ag/AgCl electrodes using an elastic cap (Electrocap), with a sampling rate of 500 Hz in a frequency band ranging from 0.5 to 100 Hz. The left mastoid was used as a reference and the right mastoid was recorded as an active channel. The data were re-referenced off-line to an average of the left and right mastoids for further analysis. The electrodes were placed according to the international 10–10 system. Vertical and horizontal electrooculogram was recorded for control of eye blinks and eye movements. Eye movement correction was accomplished by independent component analysis (Vigario, 1977). Thereafter, artifacts from head and body movements were rejected by visual inspection. Finally, data were downsampled to a sampling rate of 250 Hz, segmented in artifact free 10 s segments (i.e., comprising *N_t_* = 2500 data points each), and mean centered within segments before further analysis. Accordingly, we insured to have continuous time series of equal length for all three experimental conditions, on which multiscale analyses can be reliably applied. For the two task conditions, segments corresponded to time intervals containing a comparable number of stimuli (7–8). Table 1 shows the statistics of the resulting number of segments included in the analysis for each condition and group.

**Table 1:** Mean, SD, and minimum and maximum numbers of EEG segments per group and condition included in the analysis

		Mean	SD	Min	Max
*Young*	*Rest*	7.8	0.6	5	8
	*OnC*	23.9	2.0	15	25
	*OC*	23.0	3.1	11	25
*Old*	*Rest*	7.4	1.1	4	8
	*OnC*	22.3	3.4	12	25
	*OC*	21.6	3.1	15	25

### Metrics

Multiple metrics were applied to all data segments using MATLAB (MathWorks) or Python scripts for all calculations. We computed: the power spectrum, the spectral degrees of freedom, the detrended fluctuation analysis, the variogram, and several measures related to multiscale entropy. In general, all of these metrics relate in some way to the autocorrelation properties of the signals. However, it should be noted that neither a straightforward relationship amongst metrics, nor a direct correspondence between time scales and frequencies exist. On the one hand, the entropic measures and detrended fluctuation analysis capture nonlinear correlations in addition to linear ones, but it is not the case for the variogram and the power spectrum. On the other hand, the detrending and the coarse graining procedures (for entropic measures) transform the data in ways that make such direct correspondence impossible. In the following sections, we present the different metrics.

#### Power spectrum

For the calculation of the power spectrum (*P*), we applied a Hanning window of *N_t_* = 2500 points to each data segment. Then, after padding with trailing zeros, a 4096 point Fast Fourier Transform (using the MATLAB function *fft.m*) resulted in the complex signal in the frequency domain $X\left(k\right)=\overset{N_{p}}{\underset{j=1}{\sum }}\left(x\left(j\right)e^{\left(-2\pi i/N_{p}\right)\left(j-1\right)\left(k-1\right)}\right)$, where *x* is the signal in the time domain, $N_{p}=4096$ and indices *j* and *k* run through points in the time and frequency domain, respectively. Then, the power spectrum was calculated for positive frequencies as $P\left(k\right)=X\left(k\right)X{\left(k\right)}^{*}$, where the operator * signifies the conjugate complex number.

#### Degrees of freedom

Spectral degrees of freedom (*DoF*) is a statistic that evaluates the uniformity of spectral density (Vaillancourt and Newell, 2003). It is calculated as DoF $={\left(\overset{N_{f}}{\underset{k}{\sum }}P\left(k\right)\right)}^{2}/\frac{1}{N_{f}}\overset{N_{f}}{\underset{k}{\sum }}P{\left(k\right)}^{2}$, where *P* and *k* are as above and $N_{f}$ is the number of positive frequencies. DoF ranges from $\frac{1}{N_{f}}$ for a single peak spectral density to 1 for a completely flat one, i.e., for white noise.

#### Detrended fluctuation analysis and generalized Hurst exponent

Detrended fluctuation analysis (DFA) was introduced (Peng et al., 1994) in order to extent Hurst’s (1951) Rescaled Range Analysis for the evaluation of long-range time correlations in nonstationary signals. Its suitability for nonstationary signals has been questioned recently (Bryce and Sprague, 2012). However, it is widely used in different domains and has found many applications in biology (Hardstone et al., 2012 provides details for applications in EEG). We calculated DFA along the following steps:

1. We calculated the cumulative sum of each segment’s time series after removal of its mean: $y\left(j\right)=\overset{j}{\underset{1}{\sum }}\left(x\left(j\right)-\overset{N_{t}}{\underset{k}{\sum }}x\left(j\right)/N_{t}\right)$, where all symbols follow the above presented notation.
2. For a particular time scale *T*(*s*), with scale *s* = 4…50, and *T* = 16…200 ms in steps of 4 ms, we segmented the time series into adjacent (nonoverlapping) windows *y_ws_* of a length of *N_w_*(*s*) samples. Thus, the number of windows *W*(*s*) ranged as *W* = 625…50, and the number of samples per window as *N_w_* = 4…50, respectively.
3. For each scale s we calculated the average fluctuation across all windows as the average root-mean-square error of a polynomial fit of second order (i.e., it corresponds to removal of linear trends):
4. $\text{F}\left(\text{s}\right)=\left(\overset{\text{W}\left(\text{s}\right)}{\underset{\text{s}}{\sum }}\sqrt{1/{\text{N}}_{\text{w}}\left(\text{s}\right)\left(\overset{{\text{N}}_{\text{w}}\left(\text{s}\right)}{\underset{\text{m}}{\sum }}{\left({\text{y}}_{\text{ws}}\left(\text{m}\right)-\left({\text{a}}_{2}{\text{m}}^{2}+\;{\text{a}}_{1}\text{m}+\;{\text{a}}_{0}\right)\right)}^{2}\right)}\right)/\text{W}\left(\text{s}\right)$, where *a*_0–2_ are the coefficients of the polynomial fit, and *m* is the index of all samples within a window. We used the MATLAB functions polyfit.m and polyval.m for the calculations of the polynomial coefficients and fitting, respectively.
5. Fluctuations were plotted against time scales in a ln T(s)−ln F(s) plot and a generalization of the Hurst exponent, *H*, was calculated as the slope of the linear fit (using polyfit in MATLAB) of the resulting curve for time scales *T* in the range 24–124 msec. This range was chosen after visual inspection for linear scaling of randomly chosen data segments as well as of the groups’ mean curves for each condition. Finally, we compared both ln F(s) and *H* across groups and conditions.

*H* is indicative of the autocorrelation structure of a signal as follows: (a) for 0 < *H* < 0.5, negative correlation (anticorrelation), (b) for *H* ≈ 0.5, lack of any correlation, i.e., white noise, (c) for 0.5 < *H* < 1, positive correlation, (d) for *H* ≈ 1, 1/f or pink noise, (e) for 1 < *H* < 2, nonstationarity, (f) for *H* ≈ 1.5, brown noise. The Hurst exponent is equal to *H* for *H* < 1 and to *H* − 1 for *H* > 1 (Hardstone et al., 2012).

#### Variogram

The variogram (*V*) is an alternative way to evaluate how the magnitude of variability of a signal varies for different time scales (Cressie, 1993). However, until present its use has been limited in neurosciences (Conte et al., 2009). It has the advantage over variance in that it can be calculated for stochastic processes for which the mean is either undefined, i.e., when the related probability distribution function decays according to a power law with an exponent less than or equal to 1, or when it is hard to empirically observe, i.e., in the cases of a very large autocorrelation time. It was calculated as: $V\left(s\right)=\frac{1}{N_{s}}\left(\overset{N_{s}}{\underset{j}{\sum }}{\left(x\left(j\right)-x\left(j+s\right)\right)}^{2}\right)$, where $N_{s}$ is the number of distinct pairs of time points x(j) and x(j+s) of a distance of s samples, in the range *s* = 1…50, which corresponds to time scales T(s) in the range of 4–200 msec. Finally, we compared lnV(s) among groups and conditions.

#### Multiscale entropy measures

We calculated multiscale entropy using two different estimators: sample entropy (SampEn; Richman and Moorman, 2000), giving multiscale sample entropy (MSE), and Lempel-Ziv complexity (LZ; Lempel and Ziv, 1976), yielding multiscale Lempel-Ziv entropy (MLZ). To improve the interpretability of our results, we also estimated a normalized version of each, i.e., MSEn and MLZn (see below).

MSE was introduced by Costa et al. (2002, 2005) to evaluate the complexity of physiobiological signals, such as heart rate, i.e., the degree to which long-range correlations exist in such signals. The MSE algorithm combines the calculation of SampEn with a coarse graining procedure, acting similar, albeit not identical, to a low-pass filter, thereby precluding a one-to-one comparison between time scales and frequency content of the signal. SampEn is an improved version of the approximate entropy algorithm (Pincus, 1991), which has been designed to approximate the so-called Kolmogorov–Sinai entropy of dynamical systems (that quantifies the global temporal organization of time series and provides a meaningful index for discriminating between various dynamic systems), or the metric entropy or mean entropy rate of stochastic processes (that is, the rate with which such processes create new information), for time series of relatively short length, as it is usually the case in biology. In short, we calculated MSE along the following steps:

1. For a particular time scale *T*(*s*), with scale *s* = 1…50, and *T* = 4…200 ms in steps of 4 ms, we segmented the time series x(j)into adjacent (nonoverlapping) windows *y_ws_* of a length of *N_w_*(*s*) samples. Thus the number of windows *W*(*s*) ranged as *W* = 2500…50, and the number of samples per window as *N_w_* = 1…50, respectively.
2. We averaged all points within each window *y_ws_* to generate new time series zws=1Nw(s)∑j=1Nw(s)yws(j) for each scale *s*.
3. Then, SampEn was calculated for each of the $z_{ws}$ time series, resulting in a SampEn value for each scale, as MSE(s)=−ln(N(m+1)/N(m)), where N(m) is the number of all possible sequences of *m* points in $z_{ws}$ that are closer to each other than a distance *r*, i.e., where $\left(\left|z_{ws}\left(i\right)-z_{ws}\left(j\right)\right|<r\right)\cap \left(\left|z_{ws}\left(i+1\right)-z_{ws}\left(j+1\right)\right|<r\right)\cap .\,\;\;.\,\;.\cap \left(\left|z_{ws}\left(i+m-1\right)-z_{ws}\left(j+m-1\right)\right|<r\right)$ and i<j (no self-matches are counted). Thus, SampEn evaluates the percentage of similar sequences of *m* points that are still similar (in terms of distance) when the next point, i.e., the *m* + 1, is added to the sequence. In all our calculations we set *m* = 2 and *r* as 50% of the SD of the original signal x(j), i.e., at scale 1.

However, the SampEn algorithm has not been analytically proven to converge toward metric entropy and requires a preliminary setting of the parameter m that could lead to an underestimation if set inappropriately. We therefore also tested the LZ complexity, which is an adaptive entropy estimator. In addition to being parameter-free, it was shown to be reliable even for short sequences of a few hundreds of symbols (Lesne et al., 2009). We used the same procedure as described above, but at step 3, we calculated LZ instead of SampEn. In the LZ compression algorithm, a symbolic sequence of length *N_s_* is parsed recursively into words, considering as a new word the shortest one that has not yet been encountered. For instance, in a binary example the sequence 100110111001010001011 . is parsed according to 1 . 0 . 01 . 10 . 11 . 100 . 101 . 00 . 010 . 11 … . One then computes $LZ=N_{w}\left(1+\log_{k}N_{w}\right)/N_{s}$, where *N_w_* is the number of words used and *k* is the number of symbols in the “alphabet”. Under the assumption that the source is stationary and ergodic (assumptions that apply to the SampEn estimator as well), Lempel–Ziv theorems ensure that LZ coincides with the entropy rate up to a factor logk with$\underset{N\to \infty }{\lim}LZ=h/\log k$, where *h* corresponds to metric entropy. We used an equiquantization procedure (Hlaváčková et al., 2007) to convert signals into symbolic sequences by partitioning them into four bins (*k* = 4). The bin size was inversely proportional to the distribution of the amplitude values of EEG, such that the number of values was the same in all bins.

MSE curves have been shown to be highly influenced by the effect of the coarse graining procedure on the SD at each scale (Nikulin and Brismar, 2004). Therefore, we also calculated the SD across scales *SD*(*s*) (i.e., after coarse graining) as well as MSEn(s), for which we set a different threshold *r*(*s*) for each scale that was equal to 50% of SD(*s*) (i.e., relative to the SD of the coarse grained signal ${\text{z}}_{\text{ws}})$. This normalization was also applied to MLZ by applying at each scale a new grid, adjusted to the variance of the coarse-grained signal.

### Partial Least Squares statistical analysis

We used “*contrast*” or “*non-rotated task partial least squares*” (PLS; as implemented in MATLAB by McIntosh and Lobaugh, 2004; Krishnan et al., 2011 provides updated information) to test the main effects of groups and conditions differences. In a nutshell, contrast task PLS is a multivariate statistical method that is suitable for testing hypotheses about spatial and/or time distributed signal changes by combining information across the different signal dimensions (in our case, channels and time scales or frequencies). PLS addresses both the problem of multiple comparisons for statistical significance and of that of elementwise reliability via a permutation test and a bootstrap resampling test, respectively. A task PLS analysis with *N_g_* groups and *N_c_* conditions starts with a data matrix for each group and a contrast matrix of maximally *N_g_* × *N_c_* − 1 (as many as the degrees of freedom) orthonormal contrasts that represent the hypotheses to be tested. The rows of each data matrix contain a metric’s data points or elements of participants within conditions, which in our case were a metric’s values for all channel and time scale or frequency combinations. From those two matrices, a covariance matrix is calculated that contains the covariance of each orthonormal contrast with each element across participants. This matrix is subjected to singular value decomposition (SVD) resulting in three matrices: (1) the orthonormal matrix of the saliences of the contrasts (as determined by the initial contrast matrix) i.e., it contains the task (or design) latent variables that describe the relations among the conditions and groups of our design; (2) the orthonormal matrix of element saliences that are proportional to the covariance of each metrics’ element with each one of the task contrasts, i.e., it describes the so-called brain latent variables; and (3) the diagonal matrix of singular values that are indicative of the variance explained by each contrast. Then, a permutation test on the singular values, with resampling of the initial data matrices, results in a *p* value for each contrast tested. Finally, a bootstrap test with resampling of the initial data matrices, with replacement within conditions and groups, results in statistical reliability estimations of each element of both the task and the brain latent variables within a chosen level of confidence. Thus, the bootstrap test controls for the robustness of the results among participants. For the task latent variables, we plotted intervals of 95% confidence. Conditions with nonoverlapping intervals are robustly distinguished by the respective contrast. For the brain latent variables, we calculated bootstrap ratios by dividing each element with its SE as calculated by the corresponding bootstrap sample distribution. Bootstrap ratios >2.5758 approximate the 99^th^ two-tailed percentile for a particular element. Regarding the statistical table (Table 2), we calculated the Agresti–Coull 95% confidence intervals for the *p* value of all permutation tests, assuming a binomial distribution for the probability that a permutation sample will lead to a larger eigenvalue than the observed one (Brown et al., 2001), whereas for the bootstrap tests we direct the reader to the corresponding figures, where the confidence intervals of the task latent variables and the bootstrap ratios of the brain latent variables are depicted.

**Table 2:** Statistical table

Effect	Metric	Data structure	Type of test	Confidence intervals
Group main effect	*P*	Empirical	*permutation*	[−0.0008, 0.0046]
			*bootstrap*	Figure 5 for Task LV confidence intervals and Figure 6 for the Brain LV bootstrap ratios
	ln(*V*)		*permutation*	[−0.0008, 0.0046]
			*bootstrap*	Figure 5 for Task LV confidence intervals and Figure 6 for the Brain LV bootstrap ratios
	SD		*permutation*	[−0.0008, 0.0046]
			*bootstrap*	Figure 5 for Task LV confidence intervals and Figure 6 for the Brain LV bootstrap ratios
	ln(*F*)		*permutation*	[−0.0008, 0.0046]
			*bootstrap*	Figure 5 for Task LV confidence intervals and Figure 6 for the Brain LV bootstrap ratios
	DoF		*permutation*	[−0.0008, 0.0046]
			*bootstrap*	Figure 5 for Task LV confidence intervals and Figure 7 for the Brain LV bootstrap ratios
	*H*		*permutation*	[−0.0008, 0.0046]
			*bootstrap*	Figure 5 for Task LV confidence intervals and Figure 7 for the Brain LV bootstrap ratios
	MSE		*permutation*	[0.0000, 0.0078]
			*bootstrap*	Figure 5 for Task LV confidence intervals and Figure 8 for the Brain LV bootstrap ratios
	MSEn		*permutation*	[−0.0008, 0.0046]
			*bootstrap*	Figure 5 for Task LV confidence intervals and Figure 8 for the Brain LV bootstrap ratios
	MLZ		*permutation*	[−0.0008, 0.0046]
			*bootstrap*	Figure 5 for Task LV confidence intervals and Figure 8 for the Brain LV bootstrap ratios
	MLZn		*permutation*	[−0.0008, 0.0046]
			*bootstrap*	Figure 5 for Task LV confidence intervals and Figure 8 for the Brain LV bootstrap ratios
Condition main effect	*P*		*permutation*	[−0.0008, 0.0046]
			*bootstrap*	Figure 9 for Task LV confidence intervals and Figure 10 for the Brain LV bootstrap ratios
	I*n*(*V*)		*permutation*	[−0.0008, 0.0046]
			*bootstrap*	Figure 9 for Task LV confidence intervals and Figure 10 for the Brain LV bootstrap ratios
	SD		*permutation*	[−0.0008, 0.0046]
			*bootstrap*	Figure 9 for Task LV confidence intervals and Figure 10 for the Brain LV bootstrap ratios
	ln(*F*)		*permutation*	[−0.0008, 0.0046]
			*bootstrap*	Figure 9 for Task LV confidence intervals and Figure 10 for the Brain LV bootstrap ratios
	MSE		*permutation*	[−0.0008, 0.0046]
			*bootstrap*	Figure 9 for Task LV confidence intervals and Figure 11 for the Brain LV bootstrap ratios
	MSEn		*permutation*	[0.0000, 0.0078]
			*bootstrap*	Figure 9 for Task LV confidence intervals and Figure 11 for the Brain LV bootstrap ratios
	MLZ		*permutation*	[0.0548, 0.0865]
			*bootstrap*	Figure 9 for Task LV confidence intervals and Figure 11 for the Brain LV bootstrap ratios
	MLZn		*permutation*	[0.0018, 0.0120]
			*bootstrap*	Figure 9 for Task LV confidence intervals and Figure 11 for the Brain LV bootstrap ratios

In our design, we had two groups (i.e., *N_g_*= 2), namely Y and old O participants, and three conditions (*N_c_*= 3), i.e., R, OnC, and OC as explained above. We tested two orthogonal contrasts. The weights for the first one before normalization were set to 1 for Y-Rest, Y-OnC, and Y-OC, and to −1 for O-R, O-OnC, and O-OC, i.e., the main group effect (Y-O). Similarly, the weights for the second contrast were set to 1 for Y-R and O-Rest, 0 for Y-OnC, and O-OnC, and −1 for Y-OC and O-OC, i.e., the main effect of conditions that orders them from the task requiring the least attention and effort (Rest) to the one demanding the most (OC). Our choices for these contrasts were hypotheses driven, and as such, they have clear interpretations. However, they were also justified to a large degree in terms of the amount of variance in our data that they actually explain. We confirmed this by running an alternative explorative version of task PLS, namely a “mean-centering task PLS”. Following this version of the method, not only the brain latent variables but also the task ones are allowed to “rotate” during the SVD of the mean-centered and concatenated auto-covariance matrix of the initial group data matrices, to explain as much variance of the data as possible (always under the constraint of orthogonality; McIntosh and Lobaugh, 2004 shows a detailed description of the method). For all metrics, the first two latent variables of the mean-centering task PLS corresponded to contrasts similar (albeit not identical) to the ones we tested (group and condition main effects), and explained approximately 77–99% and 1–15% of the total variance, respectively, and 88–99% in sum.

## Results

**Figure 1:**
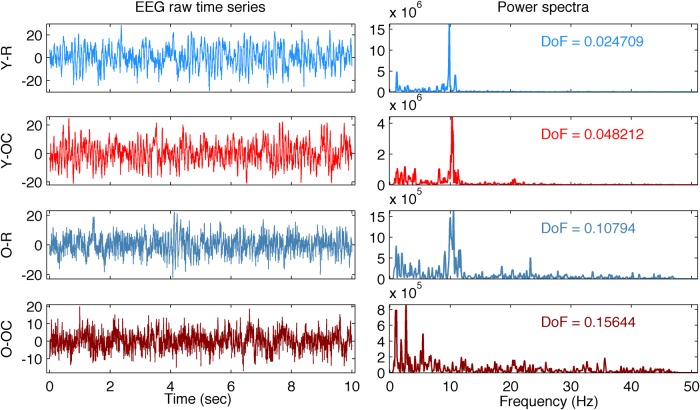
EEG time series and power spectra of randomly chosen data segments. Time series (left column) and power spectra (right column) of randomly chosen data segments for channel Cz of two participants, one young and one old, are shown. R condition is presented in in blueish colors, and OC in reddish colors. From top to bottom, the two conditions for the young participant, and then similarly for the old one. The corresponding DoF is reported with each power spectrum. A peak close to 10 Hz is apparent in all cases but for the OC condition of the old participant.

**Figure 2:**
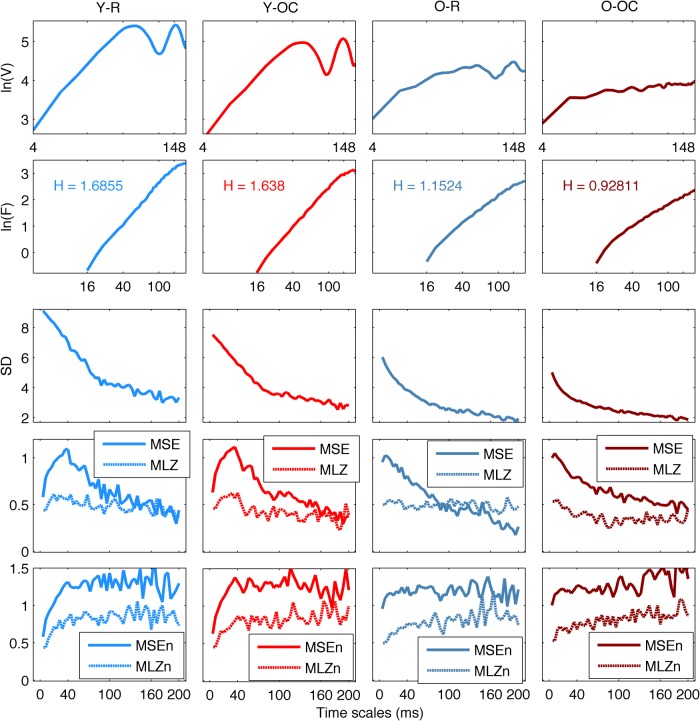
Multiscale metrics of randomly chosen data segments. The multiscale metrics of the same data segments of Figure 1 are shown, the metrics being arranged from top to bottom ln(*V*) and ln(*F*), also depicting the value of *H*, in logarithmic scale, then, SD, MSE (solid line), and MLZ (dotted line), and finally, SEn (solid line) and MLZn (dotted line), in linear scale, and data segments arranged from left to right column, in the same colors as in Figure 1. The frequency peaks close to 10 Hz correspond to local minima of ln(*V*) at the time scale of 100 ms. The peaks of MSE and MLZ, as well as the first peaks of the ln(*V*), close to the time scale of 40 ms, are related to the fact that most of the power of the signals lies below 50 Hz. Accordingly, the instances where ln(*V*) reduces again after the time scale of 148 ms correspond to additional power peaks in the low theta and delta frequencies. However, in general, there is no straightforward relationship between frequencies of the power spectra and time scales of the metrics that undergo either detrending ln(*F*) of DFA or coarse graining (SD, MSE, MLZ, MSEn, and MLZn).

To give the reader an intuition on the metrics and their comparability, as well as some guidance in the interpretation of the results, we illustrate in Figures 1 and 2 representative EEG traces and their respective metrics curves. Figure 1, left column, depicts randomly selected data segments from two participants, one young and one old, for the resting state, requiring the least attention, and the Oddball counting, requiring the most attention, conditions. In Figure 1, right column, the corresponding power spectra (*P*) and the associated DoF are shown. The results of the respective mutliscale metrics are presented in Figure 2. In the following sections, we report the observed effects with respect to aging and experimental conditions for all the different metrics.

### Between group differences: aging effects

**Figure 3:**
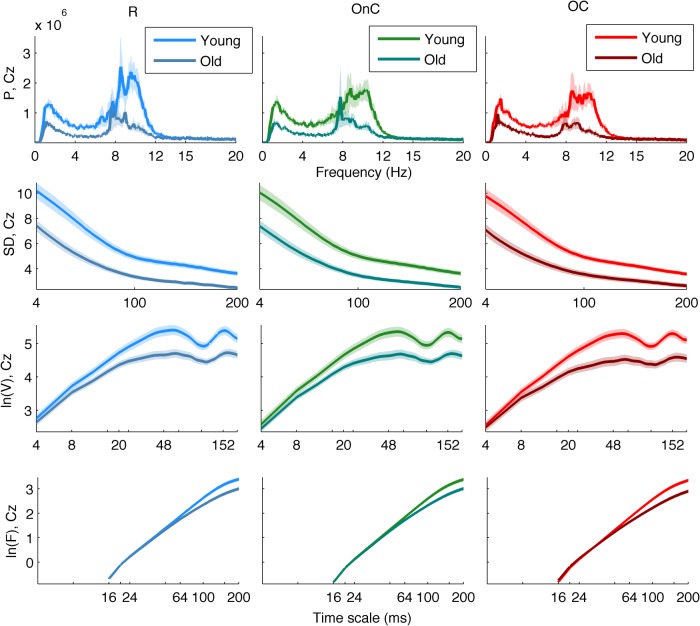
Group means and SE intervals of the metrics of the variability magnitude across conditions. From top to bottom: P, ln(*V*), SD, and ln(*F*) are shown for channel *Cz,* for all conditions (R, bluish colors; OnC, greenish colors; OC, reddish colors, from left to right columns), with darker colors for old participants (lighter for young). Thick lines and areas of faded colors represent the means and the SE intervals, respectively. Horizontal axes depict frequency for P, and time scale logarithmically for ln(*F*) and ln(*V)*, and linearly for SD. Please note the group differences, which are similar (but not identical) among conditions. The magnitude of variability is generally higher for young participants than old participants across scales, particularly so for longer time scales and lower frequencies (except for a small interval ∼8 Hz).

**Figure 4:**
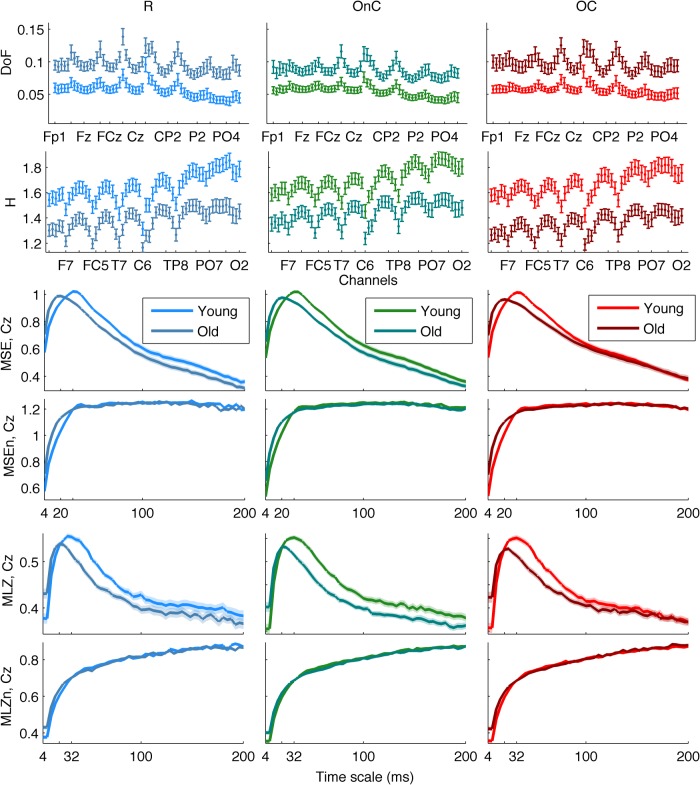
Group means and SE intervals of the metrics of the of variability structure across conditions. From top to bottom: DoF, generalized *H*, MSE, MSEn, MLZ, and MLZn for all conditions. The arrangement of columns, as well as the color and line conventions, are similar to Figure 1, except for DoF and *H*, where error bars are used to depict the standard error intervals. For DoF and *H*, all channels are shown along the horizontal axis (from frontal to occipital and left to right hemisphere ones), whereas channel Cz is shown for the rest of the metrics. Thus, the horizontal axes for those metrics depict time scale in a linear scale. Please note the group differences, which are similar (but not identical) among conditions. In particular, DoF are more and *H* is lower for the old participants than the young participants across all channels, MSE and MLZ are higher for old participants for short time scales, <24 and 20 ms, respectively, and the inverse for longer scales. MSEn and MLZn are also higher for old participants for scales <32 ms, but the effect for longer time scales is weaker.

We first investigated group differences between young and old participants by performing a separate contrast task PLS analysis for each metric for the main effect Y – O. Group differences can be inspected in Figures 3 and 4, where the mean values with SE intervals are depicted. The Cz electrode was chosen to visualize mean differences because oddball responses are well represented by the central electrodes (Müller et al., 2008, 2009), and generally Cz is less affected by muscle artifacts. The permutation tests showed that the contrast was significant for all metrics (*p* < 0.001, except for MSE, for which *p* = 0.002). These effects were to a large degree homogeneous among conditions (albeit not identical), and statistically reliable according to the bootstrap tests as shown in Figure 3. In the following we describe the main patterns of the results via the mean and SE intervals (Figs. 3 and 4), and the bootstrap ratios of the brain latent variables (Figs. 6–8). As regards the magnitude of variability metrics (Figs. 3 and 6), it can be seen that the young participants had reliably more power (P) at frequencies below 12 Hz (with the exception of a narrow band around 8 Hz), as well as a larger magnitude of detrended fluctuations [ln(*F*)], variance [ln(*V*)], and SD. The effects of the last three metrics were generally reliable across channels and scales, although they were the strongest for the parieto-occipital channels and longer time scales. As for the metrics that evaluate the structure of EEG variability across time scales (Figs. 4, 7 and 8), the elderly’s degrees of freedom of all channels’ power spectra were larger than that of the young participants, i.e., the former’s spectra were flatter. Moreover, the DoFs were the highest for the anterior channels, as well as for the lateral ones, which were also noisier (Fig. 4). Figures 4 and 7 also show that the magnitude of the detrended fluctuations coincided with a larger Hurst exponent for young participants than for the older. On average, *H* was around 1.5 for older participants and 1.7 for the young one (Fig. 3). In both groups, *H* values were the highest for more posterior, as well as midline (and also less noisy), channels. With respect to the entropic metrics (Fig. 8), relative to the young participants, entropy was higher for the older participants at time scales shorter than 24 ms, and lower at longer scales from this point on. Exemplified in Figure 4, the MSE curves of channel Cz across all conditions show a crossing point. The effect below the crossing point (i.e., higher entropy for the old participants for short time scales) was slightly stronger at the parieto-occipital channels, whereas the effect above the crossing point (i.e., higher entropy for the young participants for long time scales) was stronger at the frontocentral channels, and was present at least up to the scale of 80 ms (Fig. 8). After normalizing for the SD at each scale after coarse graining, the resulting MSEn also showed group differences, but in this case mainly so for time scales lower than 32 ms, where SampEn was higher for old participants. In contrast, the differences between groups for longer time scales were not as strong. Results were similar for the Lempel-Ziv entropy metrics (MLZ and MLZn) shown in Figures 4 and 8. However, effects were statistically weaker than for MSE, and the crossing point tended to be one scale shorter for *MLZ*, i.e., at 20 ms, and one scale longer for MLZn, i.e., at 36 ms.

In summary, the metrics that primarily evaluate the magnitude of variability across scales (the power spectrum, the detrended fluctuations’ amplitude, the variogram, and the SD), indicated that the young participants exhibited larger fluctuations, mainly so for low-frequencies, long time scales, and for the posterior channels. Inversely, entropy differences between groups reversed at the scale of 20–24 ms, and showed higher entropy for old (young) participants at shorter (longer) time scales, mainly so for posterior (anterior) channels, respectively. Normalizing for the standard deviation after coarse graining substantially weakened the effect at the long time scales. The generalized Hurst exponent, as a metric of complexity (or structure in the variability), was in accordance with the SampEn at long-scales, which was higher for the young participants, whereas the more DoF of the old participants was to be expected given their “flatter” power spectrum, especially for the lower frequencies <12 Hz.

### Effects of experimental conditions

**Figure 5:**
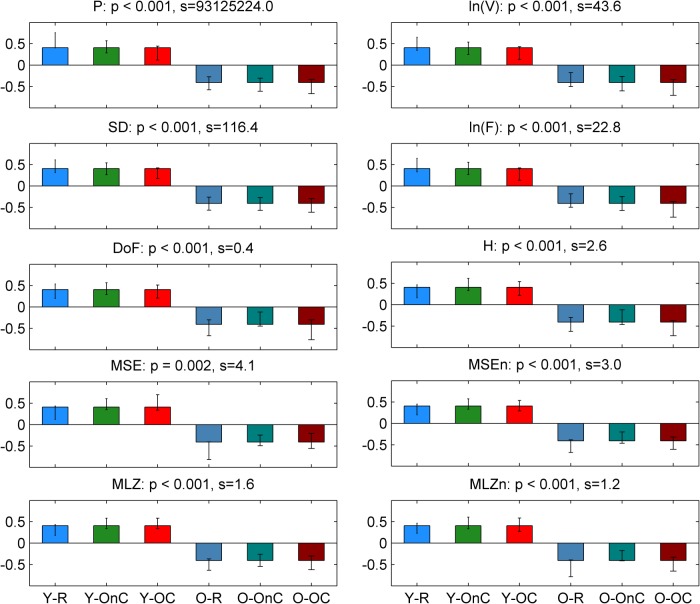
Task latent variables for the group main effect. Each panel shows the weights of the task latent variables of the contrast that corresponds to the group main effect Y-O. Each bar corresponds to a group-condition combination, with groups being arranged in increasing age from left to right, and conditions arranged in an order of increasing attention and/or task demands (i.e., from R to OC), also from left to right. Color conventions are identical to previous figures. The name of each metric together with the corresponding *p* value (as derived from the parametric test for significance) and the singular value *s* of the SVD (proportional to the variance explained by the contrast) are shown on top of the respective panel. Nonoverlapping confidence intervals signify that conditions and/or groups are separated reliably by the contrast. Thus, the contrast is significant and reliably separates the two groups.

We next tested for the main effect of condition, mainly contrasting R and OC, as the OnC was placed in the middle. The permutation test showed that the contrast was significant with *p* < 0.001 for *P*, ln(*V*), SD, ln(*F*), and MSE, and with *p* = 0.002 for MSEn, *p* = 0.069 for MLZ, and *p* = 0.005 for MLZn. The contrast was not significant for DoF and *H* (*p* > 0.1). Notably, the contrast for condition explained much less variance in our data than that for group, which was revealed by comparing the singular values of the condition contrast for each metric in Figure 8 with the corresponding ones for the group contrast in Figure 5 (the latter were much larger). As further illustrated in Figure 8, the bootstrap test showed that for *P*, ln(*V*), SD, and ln(*F*) the three conditions could not be separated reliably with a confidence of 95% (the respective confidence intervals around the weights of the task latent variables were largely overlapping). Instead, for the entropic metrics (MSE, MSEn, MLZ, and MLZn) R was generally reliably separated from the task conditions (OC and OnC), which was more clearly so for young participants. To evaluate the statistically reliable effects, as well as the statistically un- or less-reliable tendencies, we here present the brain latent variables for all metrics (Figs. 10 and 11). As for the metrics of the magnitude of variability, ln(*V*) and ln(*F*) were generally higher for the resting condition across all scales and channels, but particularly so for parieto-occipital channels. The SD was higher also for R at time scales up to 100 ms, also particularly for the posterior channels. Regarding *P*, R had more power in the 5–10 and 15–30 Hz frequency intervals than the task conditions, whereas OC had more power in the delta band (i.e., 1–4 Hz), particularly so for frontocentral channels. Regarding the entropic metrics shown in Figure 11, MSE was higher (lower) for R than for OC for time scales shorter (longer) than 44–48 ms, respectively. The effect below those scales was stronger for the frontocentral channels. The result for MLZ was very similar for short scales, but was statistically weaker, and with the crossing point moving at shorter scales (32–36 *ms*). However, above the crossing point, i.e., for long time scales, the effect was practically lost. In addition, MSEn and MLZn were generally higher for R across all scales, mainly so for the frontocentral channels. This effect was statistically much stronger for the time scales <56 ms, and more generally for MSEn compared with MLZn.

**Figure 6:**
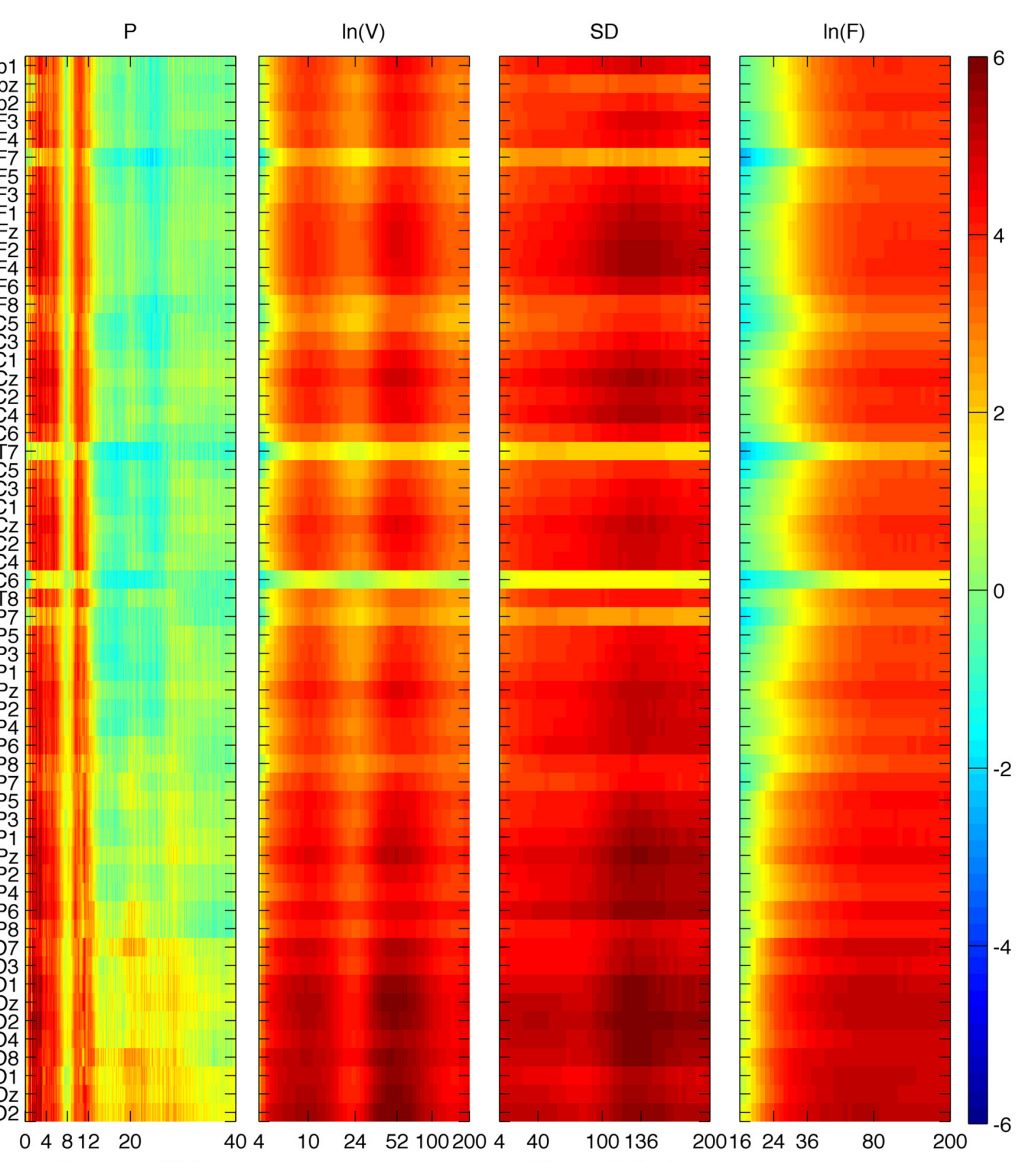
Brain latent variables for the group main effect of the magnitude of variability metrics. The panels show how much each data element, i.e., a metric’s data point, covaries with the contrast that corresponds to the group main effect (Figure 3), in terms of bootstrap ratios, from left to right: ln(*V*), SD, and ln(*F*). Absolute values >2.5758 approximate the 99^th^ two-tailed percentile. The vertical axis for all panels depicts channels arranged from top to bottom, starting from frontal and left hemisphere channels, to occipital and right hemisphere ones. The horizontal axes depict frequency for *P* and time scale in a logarithmic scale for ln(*V*) and ln(*F*), and in a linear one for SD. Because the contrast is Y-O, positive values in reddish colors signify points where young (old) participants had higher values, and the inverse for negative/bluish values. All metrics are higher for young participants: *P* for frequencies <12 Hz with the exception of a short band around 8 Hz, ln(*F*) for time scales longer than 20–32 ms, ln(*V*) for almost all time scales, and SD for all time scales. In general, the effect is statistically stronger for parieto-occipital channels, and for longer time scales and lower frequencies.

**Figure 7:**
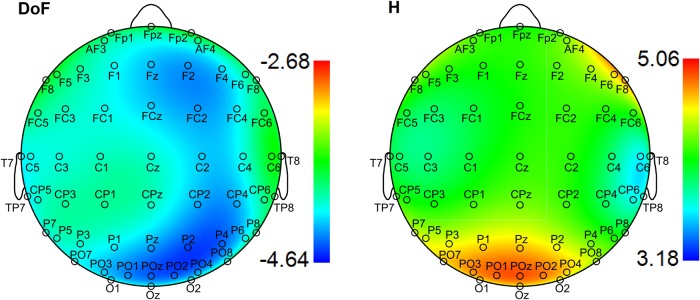
Brain latent variables for groups’ main effect for DoF and *H*. The two panels depict the bootstrap ratios of the group main effect for DoF and *H* (left and right, respectively) across a whole brain with the nose at the top. Interpretations are the same as in Figure 4. The old participants showed reliably more DoF and lower *H* for (almost) all channels than the young participants.

**Figure 8:**
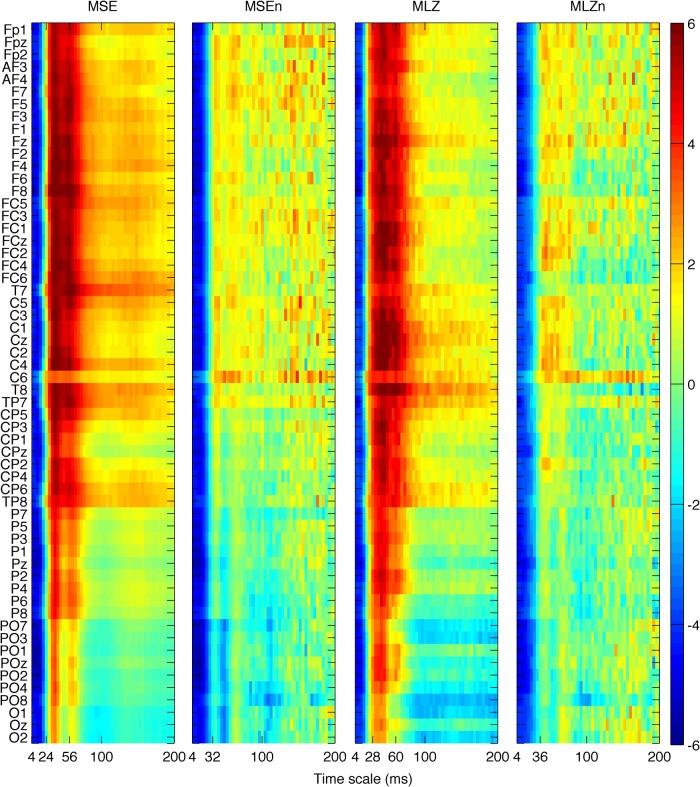
Brain latent variables for groups’ main effect of metrics of the structure of variability. The panels depict the bootstrap ratios of the group main effect for MSE, MSEn, MLZ, and MLZn, from left to right. Interpretations, vertical axes, and color conventions are the same as in Figure 4. The horizontal axes depict time scale in a linear scale. All metrics are higher for old participants below some scale (approximately 24, 32, 20, and 36 ms, respectively); this effect is statistically stronger for parieto-occipital channels. Above these scales, MSE and MLZ are higher for young participants than for the old participants up to at least the scale of 80 ms. This effect is marginally reliable for MSEn and MLZn, and stronger for frontocentral channels.

**Figure 9:**
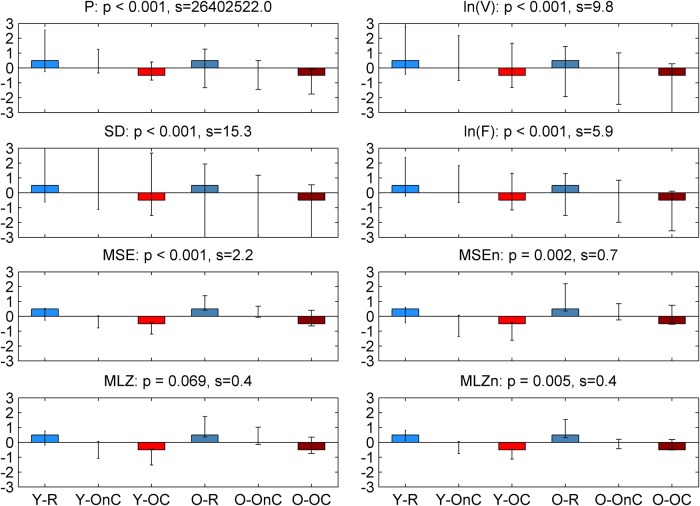
Task latent variables for condition main effect. This figure has an identical arrangement and conventions as Figure 3 (DoF and *H* are omitted because they were not significant). This latent variable contrasts R versus OdC with OnC being in the middle, i.e., it arranges conditions in an order of increasing attention and/or task demands. It is significant for almost all metrics with a *p* < 0.001, except for MSEn and MLZn that have slightly higher values (*p* = 0.002 and *p* = 0.005, respectively), whereas MLZ is significant only to a value of *p* = 0.069. However, confidence intervals are largely overlapping, i.e., conditions are not separated reliably, except for the entropic measures (MSE, MSEn, MLZ, and MLZn), where R is generally separated reliably from the task conditions, mainly so for young participants.

**Figure 10:**
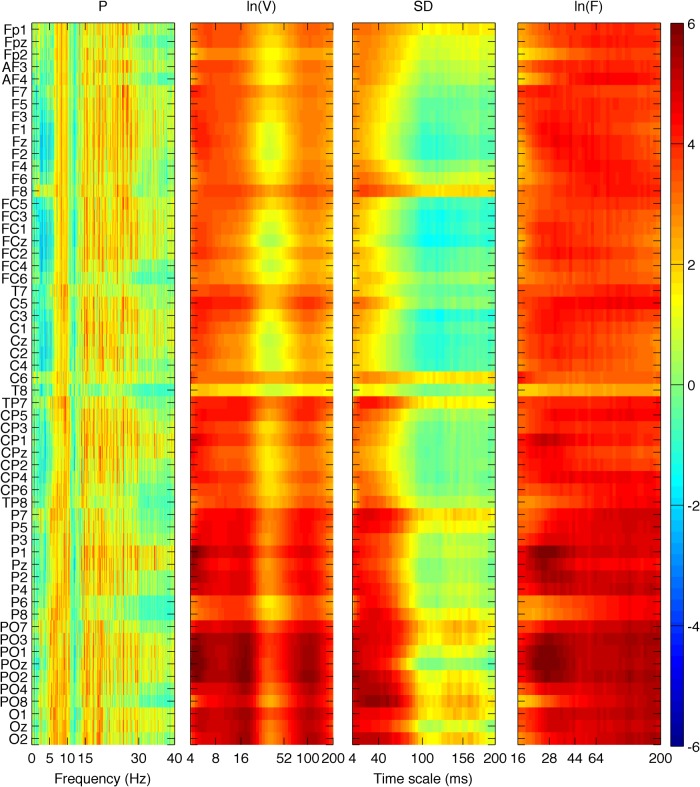
Brain latent variables for the condition main effect of the variability magnitude metrics. The panels depict the bootstrap ratios of the condition main effect for *P*, ln(*V*), SD, and ln(*F*), from left to right. Interpretations, axes, and color conventions are the same as in Figure 4, only now positive (negative) values in reddish (bluish) colors signify values that were higher for condition R (OC). ln(*V*) and ln(*F*), were generally higher for the resting condition across all scales and channels, but mainly so for parieto-occipital channels. SD was higher also for R for shorter time scales up to 100 ms, also mainly for posterior channels. Regarding *P*, R had more power in the 5–10 and 15–30 Hz frequency intervals, whereas OC had more power in the delta band, mainly so for frontocentral channels. Notice that *P* has almost an inverse pattern with the groups’ main effect in Figure 4.

**Figure 11:**
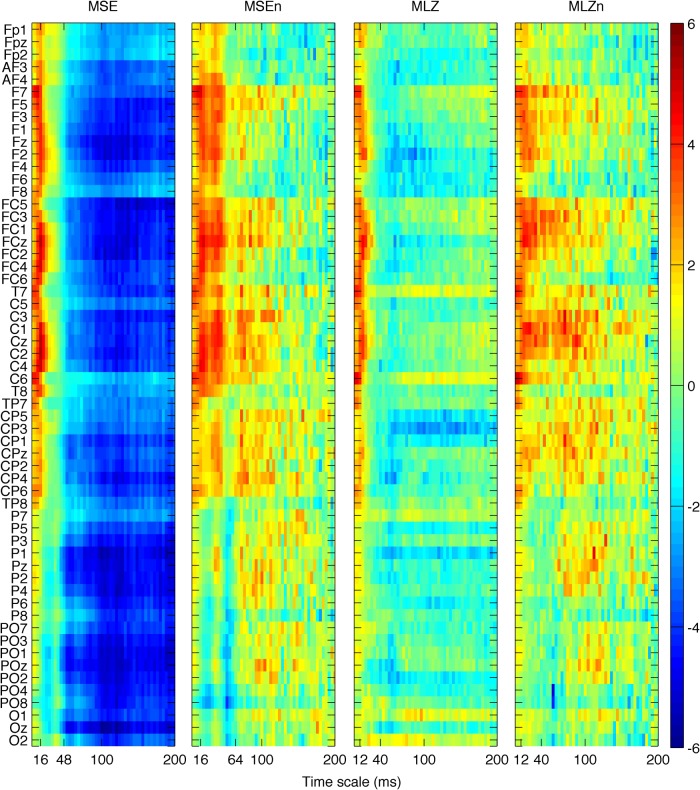
Brain latent variables for conditions’ main effect for the structure of variability metrics. The panels depict the bootstrap ratios of the condition main effect for MSE, MSEn, MLZ, and MLZn from left to right. Interpretations, axes, and color conventions are the same as in Figure 6, only now positive values in reddish colors signify values that were higher for condition R (OC), and the inverse for negative (bluish) values. All metrics are higher for R below some scale (∼48, 56, 36, and 56 ms, respectively) for frontocentral channels. MSE is higher for OC for scales above 48 ms for all channels, as well, whereas MSEn and MLZn showed a statistically weaker tendency to be higher for R for scales further than the points mentioned above and for almost all channels. Notice that the pattern of the results is to a large degree inverse to the results of the group main effect, mainly so for MSE and for short scales.

In summary, the resting state resulted generally in larger fluctuations (except for the SD at long time scales and power at the delta band at the frontal channels). Moreover, the resting condition exhibited higher (lower) entropy than the task condition with counting (OC) at short (long) time scales, respectively. However, after normalizing for the SD at each scale after coarse graining, this effect tended to reverse for long time scales. It is worth noticing that the patterns of results for *P* and MSE, as well as for MSEn, MLZ, and MLZn, at short scales only for the last three, were to a large degree inverse to those of the group main effect, i.e., the results for the attentive task (rest) condition followed the ones for the young (old) participants. This rough correspondence, however, reversed for the rest of the metrics, i.e., ln(*V*), SD, and ln(*F*).

## Discussion

The present study investigates the changes of cortical dynamics with aging through the use of a battery of multiscale metrics, which allows that characterize the structure and the magnitude of EEG fluctuations.

### Age-related differences in the magnitude of EEG signals variability across time scales

Our results show that the cortical activity of older participants displayed smaller fluctuations than young participants in a (close to) scale-independent manner. Consistent with previous studies (Dustman et al., 1993, 1999; Gaal et al., 2010; Müller and Lindenberger, 2012), EEG signals of the elderly generally contained less spectral power than that of the young adults. Similarly, the DFA, SD, and variogram results also indicated a decrease in the fluctuations’ magnitude with aging. Although, to our knowledge, this aspect of brain signal variability has never been explicitly addressed before in EEG recordings, it is in-line with recent fMRI studies (Garrett et al., 2013a,b), where older adults were found to display a reduction of SD BOLD signals in most brain areas (especially cortical) in both resting and task-driven states (Garrett et al., 2011, 2013a). Our study extends these observations to scalp EEG and shows that it is indeed a pervasive characteristic of the aging brain across time scales.

### Effects of aging on the organization of cortical fluctuations across time scales

In the frequency domain, older adults showed flatter power spectra with a lower alpha peak, and more spectral DoF, suggestive of increased ‘broadband’ noisiness of the cortical activity. Further, long-range autocorrelations were less present in older participants’ data (higher *H* exponent). The multiscale entropy metrics revealed a time-scale-dependence of aging effects regardless of the used estimator (SampEn or LZ) with the elderly’s EEG signals being more irregular at fine/shorter scales, and less complex at coarser/longer scales. Thus, young and old brains appear to operate at different time constants making them, under the effect of coarse graining, reach maximal entropy at different time scales. After reaching their respective peak, both young and older adults’ MSE/MLZ curves decreased; however, those of the young remained significantly higher. This loss of complexity across the long scales may be indicative of diminished global information integration with aging, because these scales relate mostly to low-frequency oscillations mediating long-range interactions. Mind, however, that the inverse does not directly apply, because the short scales enclose information about both high- and low-frequency oscillations. Furthermore, it is known also that (multiscale) entropy-based measures reflect both variance and correlation properties of time series (Costa et al., 2002, 2005). To extract variance-related changes, we compared the multiscale entropy curves (MSE, MLZ) with their normalized versions (MSEn, MLZn) and the SD curves. A crossing-over was present for the entropy metrics (regardless of the normalization), but not for SD, for which young and elderly’s curves were parallel. It is notable however, that, although the age-group differences in entropy remained mostly significant after normalization, they were substantially weakened. The normalization affected essentially the part of MSE/MLZ curves after the peak that contains the scales accounting for the autocorrelated (low frequencies) content of the signal, which actually contain the most power (∼<20 Hz).

The above results are in accordance with the current literature, and extend it with several new findings. First, we reproduced McIntosh et al.’s (2014) results and extended them to longer time scales, as well as to resting-state activity. For the first time scale, our findings (i.e., more irregularity for older adults) are consistent with those of other EEG studies using single-scale measures of complexity (Anokhin et al. 1996; Pierce et al. 2000, 2003; Müller and Lindenberger 2012). Conversely, our observations at longer scales approximate the observations of fMRI studies, in which the time resolution is much lower than in EEG. Indeed, fMRI investigations at resting state have also shown a loss of entropy with aging (Yang et al., 2013; Smith et al., 2014; Sokunbi, 2014).

Overall, we show that aging effects on cortical fluctuations are time-scale-dependent with regard to structure (i.e., less regular fluctuations at shorter scales and less complex fluctuations at longer scales), but not in terms of magnitude (i.e., a systematic reduction regardless of the time scale).

### Spatial patterns of variability changes with aging

The observed aging effects on EEG variability were rather robust for almost all channels. Nevertheless, some spatial patterns showing stronger effects for certain electrodes were distinguishable over the scalp. Notably, the posterior channels were found to display the highest young–old differences in terms of variability magnitude (across all scales), with the younger adults being furthermore variable than the elderly for these regions. This was also the case for the power spectrum, the variogram, and the DFA analyses. This spatial pattern of age-related differences in terms of fluctuations magnitude is consistent with the observation that young adults have more power in most of the frequency bands at the posterior areas (seen in our results, and previously reported by Gaal et al., 2010). Conversely, entropy-wise, young–old differences for the longer/coarser scales were stronger at the frontocentral channels, because EEG signals of the younger participants were more complex for these channels than for the occipital ones.

These results suggest that the detected anterio-posterior difference in the magnitude of group effects stems from a fronto-occipital differentiation expressed only in the younger adults’ brains. This interpretation corroborates the view of spatial dedifferentiation in the aged brain, as shown for instance in Garrett et al.’s (2013a,b) studies, wherein older adults were found to exhibit low and nearly indistinguishable levels of variability across brain structures in both resting and task-driven states.

### Differences between experimental conditions

The differences between resting and the auditory stimuli conditions (with and without counting) followed a similar pattern across metrics, with the contrast driven mainly by the difference between resting state and the cognitively most demanding oddball counting task. However, this distinction could only be made reliably through MSE, and more consistently so for young participants. The limited change between rest and task situations might be related to the fact that in all experimental conditions participants were instructed to keep their eyes closed. Eyes opening was indeed shown to significantly affect brain signals complexity elsewhere (Hogan et al., 2012; Müller and Lindenberger, 2012), as was also found in our preliminary analysis including the eyes-open condition. In addition, the cognitive task we used is not very demanding. With respect to MSE, the pattern of difference between the resting (least demanding) and oddball counting (most demanding) condition resembled the one differentiating the age groups (old vs young): the EEG of the less demanding task was more complex at shorter scales. A stronger difference was found for the frontocentral channels, most likely due to the attentional load imposed by the task. This difference was reversed at longer scales, where the OdC condition yielded the most entropic signals. To our best knowledge, this is the first time a specific MSE pattern with obvious time-scale-dependence is shown to differentiate between brain states at different cognitive loads. Nevertheless, the low differentiability between conditions in elderly was reported previously and seems to be one of the general signatures characterizing the senescent brain (Garrett et al. 2013a). This lack of specificity in the aged brain manifests itself, thus, both through a spatial (within experimental condition, as shown in the section before) and a “states” (between conditions) dedifferentiation.

### Convergence of aging theories and empirical findings

The dedifferentiation hypothesis initially introduced by Baltes and Lindenberger (1997) is repeatedly referred to in the literature to describe and explain cognitive declines with advanced age (for review, see Park and Reuter-Lorenz, 2009). Notwithstanding its initial framework (i.e., correlations between sensory and cognitive functions), dedifferentiation can be used to account for several facets of age-related changes in brain and behavior (Sleimen-Malkoun et al., 2014). In the brain, it can be seen through increased interdependence between functional domains (e.g., cognition and motor control; Schäfer et al., 2006, 2010), decreased specialization of brain regions (Park et al., 2004; Dennis and Cabeza, 2011), and more widespread activations (Reuter-Lorenz et al., 2000; Heuninckx et al., 2005, 2008). Nevertheless, neurobehavioral variability is not an outcome measure in the dedifferentiation approach and its extensions. In this regard, for a long time, the aging literature essentially focused on behavioral variability (e.g., response times) in relation to changes in patterns of brain activations (Hultsch et al., 2008; MacDonald et al., 2009), rather than on characterizing brain signals fluctuations themselves. The neural noise hypothesis (Li et al., 2000; Li and Sikström, 2002) is one of the first and most established approaches dealing with this aspect. It argues in favor of an increased random background activity in the aged CNS (referred to as neural noise), resulting in a higher intraindividual variability in performance (Li et al., 2000, 2001; Li and Sikström, 2002; Hultsch et al., 2002). Currently, it is widely recognized that the variability of brain activations in space and time is of high relevance to understand brain functioning in health (e.g., development; Vakorin et al., 2011; and normal aging; McIntosh et al., 2014) and disease (e.g., autism; Bosl et al., 2011; and Alzheimer disease; Mizuno et al., 2010). This rather recent interest succeeds a more established view in the domains of physiology and motor behavior where the loss of complexity hypothesis (LOCH) was developed (Lipsitz and Goldberger, 1992; Lipsitz, 2002, 2004). In this framework, the structure of fluctuations is considered to reflect the complexity of the underlying functional organization and interactions within and between different subsystems. The LOCH stipulates that during aging, as well as disease, there is a generic tendency toward less-complex (behavioral and physiological) outputs that could be in the direction of an increased regularity or an increased randomness (Goldberger, 1996; Vaillancourt and Newell, 2002, 2003), both supposedly indicative of a breakdown of functional synergies and a decoupling of components. The LOCH can be connected to dedifferentiation of brain activations by looking at the spatial distribution of variability and linking time scales of fluctuations to information processing in the brain. A more uniform spatial representation of variability across cortical and subcortical structures expresses the characteristic spatial dedifferentiation of the aging brain (Garrett et al., 2011). Conversely, the time scales view presumes that complexity at finer scales characterizes local processing, and may thus be related to short neural connections, whereas the coarser scales (by filtering out higher frequencies) reflect the more long-range (i.e., global) interactions, and therefore depend on longer neuronal fibers (Mizuno et al., 2010; Vakorin et al., 2011; McIntosh et al. 2014). McIntosh et al. (2014) argued in favor of this assumption and showed that scale differences observed with MSE follow closely those that can be quantified through other entropy measures that distinguish local and distributed informational exchanges (i.e., conditional entropy and mutual information).

All the aforementioned aging hypotheses could be linked to underlying alterations of neural structures and interactions, as well as dysregulation of neurotransmission, together leading to a less rich and flexible repertoire of functional synergies. Structurally and physiologically, the aging brain is known to incur changes characterized by a marginal neuronal loss (Wickelgren, 1996; Morrison and Hof, 1997; Bishop et al., 2010), but a substantial decline in the integrity of white matter (Sullivan et al., 2010; Madden et al., 2012), as well as a disruption in the synthesis of some neuro-transmitters (dopamine, norepinephrine, acetylcholine). These modifications greatly affect large-scale brain networks by disturbing interhemispheric functional connections and interactions (Duffy et al., 1996; Kikuchi et al., 2002; Langan et al., 2010), as well as somatosensory cortical inhibition (Cheng and Lin, 2013). Impaired dopaminergic neurotransmission further compromises the modulation of neural noise, which is an additional cause of inflexibility of brain activity and behavior (Hong and Rebec, 2012). The conjunction of all these alteration is most likely responsible for the observed changes in multiscale variability and activation patterns, which nicely merges with the predictions stemming from main aging theories. Indeed, although these theories were developed to cover different domains and mechanisms, they converge to describe systemic modifications characterizing the senescence process(es) in the neurobehavioral system (Sleimen-Malkoun et al., 2014). An essential current debate that needs to be settled is the relative importance of local (i.e., grey matter and neurotransmission degradation) and global (i.e., white matter degradation and demyelination) network changes, as well as the beneficial or detrimental role of stochastic components of brain dynamics (i.e., noise), and how these factors affect functional connectivity, brain signal variability, and performance. In the framework of dedifferentiation the degradation of neurotransmission is thought to reduce the signal-to-noise ratio in local networks leading to less distinct cortical representations, and potentially to less specific functional connectivity (Li and Lindenberger, 1999; Li et al. 2001; Li and Sikström, 2002). Functional connectivity and complexity are considered to entertain an inverse relationship, according to which higher entropy is found when connectivity is poor, and vice versa (Friston, 1996; Müller and Lindenberger, 2012; Ghanbari et al., 2013). However, from a different perspective, the reverse is commonly suggested (cf. Vakorin et al., 2011) based on the assumption that information processing and (neural) complexity go hand in hand (Tononi et al., 1994, 1998; Slifkin and Newell, 1999). Conversely, following the finding that neural information transmission is determined by both the degree and time scale of synchrony (Baptista and Kurths, 2008), a different view can be suggested. Accordingly, neural processing would be maximized when synchronization is high at coarse time scales (strong connectivity requiring complexity to be low) and low at fine scales (weak connectivity allowing the expression of greater complexity). Evidence for such time-scale-dependence, with a negative connectivity–complexity association at fine scales and the reverse at coarser scales was found in resting-state fMRI data (McDonough and Nashiro, 2014), as well as in mean field model and BOLD simulations (Jirsa et al. 2010; Nakagawa et al., 2013). Therefore, it could be concluded that entropic and variability changes convey different information depending on the time scale under scrutiny. More precision should be gained in the future by accounting for the recently uncovered nonstationarity of the dynamics of resting state fMRI (Allen et al., 2014), which is expressed through different functional connectivity measures for different time windows and moments in time. Hansen et al. (2015) demonstrated that the nonstationarity of the resting-state dynamics is evident in rapid changes in functional connectivity patterns, which are otherwise relatively invariant during epochs lasting one to two minutes. These transitions are reminiscent of phase transitions as known from statistical physics and were referred to as functional connectivity dynamics (FCD; Hansen et al., 2015). A successful quantification of FCD promises to provide a more profound understanding of variability- and complexity-related phenomena in brain networks, and thus ageing-related changes in brain and behavior.

Overall, it appears that although the aging brain displays more widespread activations, in terms of information processing, it is characterized by an increased spatial clustering with a shift toward a lesser contribution of long-range connections (Meunier et al., 2009). However, the contribution of changes in connectivity and nonstationaries remains to be unraveled.

## Conclusion

Our findings provide support to the importance of multiscale brain signal variability as a means to assess the effects of aging on brain functioning. Even though no absolute value or a single metric can currently be offered as a biomarker of brain age, the contribution of a systematic study of variability through multiple measures and scales rests in the link that can be established with functional and structural connectivity, as well as the richness of activation patterns. Nevertheless, we argue that any expected or discussed effect of aging should meet the complexity of the functional organization within the human neurophysiological and neurobehavioral system, which makes simple, strict, and irrevocably generalizable correspondences unlikely to be found. It would be misleading, for instance, to expect that aging is a process of “loss”, and that what is observed in term of behavior mirrors sensu stricto changes in brain activations. In the brain, what counts most to insure a rich adaptable behavior is the interplay between multiple factors, namely, local and global neuroanatomical connectivity, noise levels, and interaction delays (Ghosh et al., 2008; Jirsa et al., 2010; Deco et al., 2011). Accordingly, the healthy brain expresses critical magnitudes and structures of variability that undergo significant changes with development, aging, and disease. Regarding aging, some general features can be extracted. Mainly, a pervasive reduced level of variability, in terms of magnitude, an increased irregularity at shorter time scales, a decrease complexity at long scales, and finally a spatial dedifferentiation in activations and between brain states (e.g., rest vs task). The meaning of these changes and their link with structure, function, and dynamics can be significantly furthered and made more explicit through theoretical and simulation studies, and empirical investigations. Systematic investigation of how aging-relevant functional and structural modifications affect the outcome of multiscale variability and complexity metrics would offer a major contribution. A wider set of entropy estimators (e.g., epsilon entropy) and metrics can also be covered (multivariate measures, synchronization measures, Lyapunov exponents, etc.). However, it is to be expected that these supplementary methods will provide converging evidence in terms of global effects, as it has been found in the present study for the measures quantifying fluctuations’ magnitude and those quantifying their structure. Therefore, based on our findings we contend that adding more metrics would not profoundly advance our current understanding of aging. Conversely, a novel and more promising direction would be appropriately taking into account the nonstationary nature of brain processes, which seem to be an inherent property of brain functioning and to occur on various scales of organization (Hansen et al., 2015). Finally, combining different modalities of brain imaging and investigating different brain states in a single aging experiment would make it possible to irrefutably relate the different phenomena that have been separately shown to characterize aging (e.g., dedifferentiation, loss of complexity, variability changes), as well as integrate newly uncovered ones (e.g., nonstationaries in functional connectivity; Allen et al., 2014; Hansen et al., 2015), while establishing the link with performance and behavior.

## Figures and Tables

**Figure:**
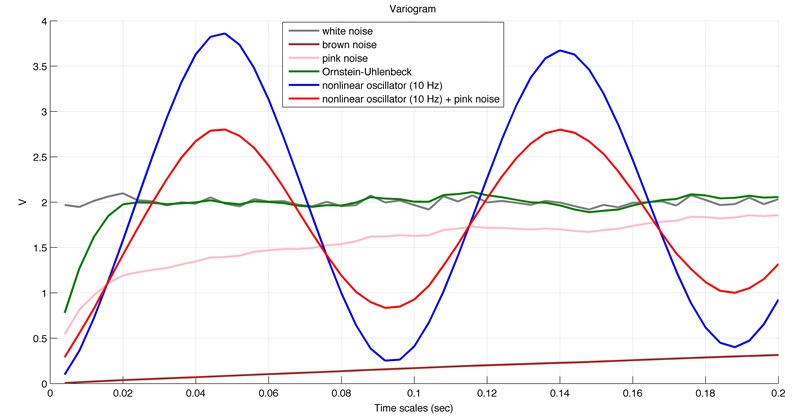


## Synthesis

The decision was a result of the Reviewing Editor Tatyana Sharpee and the peer reviewers coming together and discussing their recommendations until a consensus was reached. A fact-based synthesis statement explaining their decision and outlining what is needed to prepare a revision is listed below. The following reviewers agreed to reveal their identity: Arnold Mandell and Woodrow Shew

1) Please focus the initial exposition of results on a few measures, such as entropy and power spectra. This will improve the readability, and therefore impact, of your manuscript. One could then simply point that other measures (to be discussed in a Supplement or an Appendix) yield similar conclusions. It would also be helpful to point out if some measures that others and whether some kind of normalization would make comparisons among the measures (i.e. relative to control in a normalized setting) more meaningful.

2) Reviewer 2 makes several specific suggestions for how to improve the signal-to-noise ratio in the analysis of differences between the resting and task-related signals. These include using channels close to the auditory cortex and limiting the analysis to time intervals where the mean activity shows differences.

3) It would be better to introduce the work by considering the impact of not only noise-induced switching between stable states (as currently done in the manuscript), but also of the inherently unstable dynamics.

4) Please show raw time series and a channel map for readers not familiar with EEG. Detailed comments by the reviewers are included here:

Reviewer 1:

Since multiplicity of measures is a methodological thrust of the paper, perhaps the authors should consult the work of Ornstein, Adler, Weiss and other ergodic theorists of dynamical systems who have proven that in these kind of complex systems, there is only one non-redundant measure, the entropy. Thus the agreement among measures for given time and space scales and experimental conditions shouldn't be surprising. I found the self congratulatory uniqueness claimed by the authors was not deserved and a bit off putting. Otherwise, it is a solid piece of work that should see the light of day.

Reviewer 2:

The authors address two questions: 1) whether fluctuations of EEG measurements systematically change with age, and 2) whether fluctuations of EEG change between when a subject is resting or performing an auditory oddball task. The authors employ multiple measures to assess the character of fluctuations at different timescales and, to a lesser extent, different spatial scales. The authors demonstrated convincingly that EEG fluctuations do indeed change with age. However, their work on how EEG fluctuations change depending on resting state versus task conditions was less convincing.

The meaning and function of fluctuations in cortical dynamics is an important topic in systems neuroscience. However, it was already known that fluctuations of brain dynamics depend on age (the authors point this out and cite many previous studies). Therefore, this work represents a marginal advance compared to previous findings. The authors' experimental design had the potential to result in a more substantial advance related to the difference between resting state and task related fluctuations, but this potential was apparently not realized.

MAJOR CONCERNS

1. The authors employ too many different measures of fluctuations and entropy. It seems to me these different measures resulted in largely overlapping findings. However, the findings were presented in such a way that all the different measures have equal importance. The result of this style of presentation is that the main points are severely diluted with confusing, redundant, and ultimately unimportant repetition. The entire paper reads like the 'Supplementary Material' section of a more succinct paper. In other words, a reader puts significant effort into understanding the meaning of all the different metrics, but, in the end, it would have sufficed to only know about power spectra and MSE. By the end of the paper, the effort a reader puts into understanding all these metrics seems to have been a waste of time, because most measures do not provide extra insight. This problem could be remedied if the authors put a major effort into distilling out the main point with the few measures that revealed important points. The rest of the measures should be relegated to some later figures that a reader need not read unless he/she wants to read a lot of detailed supporting, redundant information.

2. The authors do not present any raw data. It would be very helpful if the authors could show some raw EEG traces that show qualitatively how the fluctuations change from young to old subjects. Is there any apparent difference between a timeseries with high MSE compared to one with low MSE? Likewise, it would be nice to see some evidence that the task results in some trial-averaged response that is measureable with EEG probes. This is necessary to establish the extent to which the task results in some deviation from resting state activity.

3. The authors did not sufficiently explore the task/stimulus evoked EEG. The authors report a small small but significant difference between resting and task EEG fluctuations, but they did not present a convincing argument that they treated the task/stimulus evoked data appropriately. For example, it would be very sensible to first use some kind trial-averaged evoked timeseries to identify the time period following the oddball stimulus that results in a significant average response. Then after establishing such a "response time period", they could examine fluctuations within the same time period. Moreover, if the authors presented such trial-averaged EEG signals, this would give more convincing evidence that the experimental task has some measureable effect on the EEG signals. Finally, it would probably make the most sense to seek task related changes on an EEG channel near auditory cortex rather than channel Cz (a channel map should be provided in the manuscript for readers without expertise in EEG). As far as I can tell from the methods, the authors simply divide up the data into 10 s segments regardless of when the auditory stimulus is delivered. In my view, this lack of effort to identify the evoked response makes it somewhat ambiguous whether or not there is a significant difference in the variability of stimulus evoked response for young vs. old.

MINOR POINTS

pg 1 lines 9-11: This sentence sounds like the age-related changes only occurred in time-scale dependent experimental conditions. Consider rewriting in two sentences for clarity.

pg 2 lines 4-13: This seems like a 'pet theory' that is rather disconnected from the experimental data presented. It is better suited to the discussion section. Line 14 picks up with actual experiment facts that may be better suited to introduce the paper. Indeed, another theoretical perspective posits that there are in fact no stable states and that the system 'noise' is inherent to the dynamics as in chaotic or critical network dynamics. How does one distinguish between the situation A (stable states + noise) and situation B (inherently unstable dynamics). Perhaps this is a discussion point, but the intro should not be written as if situation A is the only possible underlying hypothesis.

pg 5 lines 5-8: It appears that your variable names are mixed up here. For example, if each time series has 2500 points sampled at 4 ms intervals, then for T=16 ms, the number of samples per window should be Nw=4 and the number of windows should be W=625.

pg 5 line 26: Was the 'variogram' developed by the authors or has it been used in previous work? Please cite it if it was used previously or state that it is a new metric if that is the case.

pg 5 line 29: This should be, "... decays according to a power-law with exponent lower or ...". Other types of decay can have well defined mean.

pg 5 line 33: Some intuition for interpreting the variogram would be nice. For example, how does it behave for white noise, pink noise, periodic signals?

pg 6 line 8-9: Again, the W and Nw variables are switched.

Figure 1:

Although the differences across groups (young vs. old) are clear, I have some concerns about interpreting the various measures that are presented in this figure. The power spectra clearly exhibit a peak around 10 Hz, which suggests that fluctuations at a time scale around 100 ms should be large. This seems to be at odds with the various other measures of fluctuations versus time scale. For example, there is no peak in SD around 100 ms. In fact, there is a decrease in ln(V) near 100 ms. These basic observations make it somewhat confusing to interpret the meaning of these measures. It would helpful if the authors could explain this.

It would be also be helpful for understanding how the measures in the bottom three rows of Fig 1 are related if the horizontal axes were aligned and all plotted in the same way (i.e. all logarithmic or all linear).

It would also be nice to see some basic time series of the data. Jumping immediately into presenting the results of these complicated measures without ever seeing the data limits the understanding and interest of the reader.

**Author Response**

**eNeuro – Response letter**

Manuscript Number: *eN-NWR-0067-14*

Article Title: *Brain Dynamics of Aging: Multiscale Variability of EEG Signals at Rest and during an Auditory Oddball Task*

**Reviewer 1:**

**The convergence of measures explained by the explanatory value of entropy in dynamical systems**: “*Since multiplicity of measures is a methodological thrust of the paper, perhaps the authors should consult the work of Ornstein, Adler, Weiss and other ergodic theorists of dynamical systems who have proven that in these kind of complex systems, there is only one non-redundant measure, the entropy. Thus the agreement among measures for given time and space scales and experimental conditions shouldn't be surprising.*”

The reviewer made a good point in this comment. Indeed, in the ergodic theory framework, entropy (or more precisely, the Kolmogorov-Sinai entropy) has been demonstrated to be an invariant measure in dynamical systems. We now make reference to this work in the Introduction (p.2) and cite the papers of Adler and Weiss (1967) and Ornstein and Weiss (1991).

Based on the theoretical results showing the non-redundancy of entropy from a general perspective, one could infer its high explanatory power. However, in empirical continuous data, generated assumingly by high-dimensional non-stationary stochastic processes, the estimation of entropy is a matter of debate. Indeed, the violation of some of the basic assumptions, such as stationarity, or the finite size effect, poses serious difficulties for an accurate estimation. Therefore, the multiplicity of metrics could be expected to result in some complementary information and the convergence of related metrics will increase the confidence of the findings. In our paper, we made the choice of testing an ensemble of metrics, which relate to those that are currently applied and developed in the context of the study of neuro-behavioral variability (e.g., variogram, DFA, MSE). It is one of the main objectives of this study to show that the basic results are robust amongst the metrics and to improve their interpretability via the sketching of their relationships when applied on the same dataset.

As pointed out by the reviewer, our results have shown that the measures of entropy overlap and could be strongly related to findings of other (variance and frequency-based) metrics. This point is now explicitly mentioned in the Discussion section along with the ceiling-effect that the addition of measures can show (see p.16).

**The contribution of the paper**: “*I found the self congratulatory uniqueness claimed by the authors was not deserved and a bit off putting. Otherwise, it is a solid piece of work that should see the light of day.*”

We apologize if the way in which our paper was written, left an off-putting impression on the reviewer. Indeed, we do not wish to evoke such an impression. We made an honest effort in the revision to correct this style and clarify our objectives (see Abstract, Significant Statement and Discussion sections).

**Reviewer 2:**

MAJOR CONCERNS

**The redundancy of metrics**: “*The authors employ too many different measures of fluctuations and entropy. It seems to me these different measures resulted in largely overlapping findings. However, the findings were presented in such a way that all the different measures have equal importance. The result of this style of presentation is that the main points are severely diluted with confusing, redundant, and ultimately unimportant repetition. The entire paper reads like the 'Supplementary Material' section of a more succinct paper. In other words, a reader puts significant effort into understanding the meaning of all the different metrics, but, in the end, it would have sufficed to only know about power spectra and MSE. By the end of the paper, the effort a reader puts into understanding all these metrics seems to have been a waste of time, because most measures do not provide extra insight. This problem could be remedied if the authors put a major effort into distilling out the main point with the few measures that revealed important points. The rest of the measures should be relegated to some later figures that a reader need not read unless he/she wants to read a lot of detailed supporting, redundant information.*”

We agree with the reviewer that the method and results section are dense and may be hard to follow, especially if one is not familiar with the different metrics.

A key objective of this work is to test a wide set of measures of multiscale EEG fluctuations as they are used in the literature, and, by doing so, provide a guide for future studies. By reducing the present work to just *Power spectra* and *MSE* the paper would miss its purpose. This central point is now made clearer in the revised version (see Abstract p.1, Introduction p.2 and Discussion p.12).

We assert that although most of the metrics we used have been reported elsewhere, they have been used separately, and for some of them, their suitability was criticized. The contribution of our study is to shed light on these aspects by showing which measures are comparable, and whether methodological limitations bias the observed results. In this respect we show that, overall, results converge between the metrics reflecting the magnitude of fluctuations and those reflecting their structure, and that using more specific measures (e.g., *Variogram*, *MSEn*, *MLZ* and *MLZn*) confirms the knowledge that can be established with more classical analysis (e.g., power spectrum and *MSE*).

Finally, a major issue, which both the reviewer and the editor seem to have missed, is the fact that eNeuro does not accept supplemental material or appendices. Therefore, we were, and still are, constrained to provide all information within the manuscript. This does not imply, however, that the readability of the paper could not be improved. Accordingly, we made the effort of reducing the presentation of redundant measures in the Methods and Results sections. Along these lines, we attempted to address the reviewer’s concern and have merged entropy measures in one section (see Metrics p.6,7), and discussed their results more concisely (see Results p.10,11).

**Visualization of changes in EEG signals**: “*The authors do not present any raw data. It would be very helpful if the authors could show some raw EEG traces that show qualitatively how the fluctuations change from young to old subjects. Is there any apparent difference between a timeseries with high MSE compared to one with low MSE? Likewise, it would be nice to see some evidence that the task results in some trial-averaged response that is measureable with EEG probes. This is necessary to establish the extent to which the task results in some deviation from resting state activity.*”

We understand that it would helpful to visualize representative time-series. We draw the attention of the reviewer to the fact that all the different metrics that are used in the paper surely afford a more robust picture of task- and age-related changes than the one that can be drawn from visual inspection. Indeed, the purpose of our analyses was to quantify the changes in EEG signals and make them more explicit and informative. We agree that this point could be made even stronger by showing time-series. Therefore, we added representative time-series of one trial from channel Cz for a young and an older participant at rest and during the oddball counting condition, as well as the results of the different metrics (see Fig. 1 and 2 of the revised manuscript).

**Task-evoked changes in averaged EEG responses**: “*The authors did not sufficiently explore the task/stimulus evoked EEG. The authors report a small small but significant difference between resting and task EEG fluctuations, but they did not present a convincing argument that they treated the task/stimulus evoked data appropriately. For example, it would be very sensible to first use some kind trial-averaged evoked timeseries to identify the time period following the oddball stimulus that results in a significant average response. Then after establishing such a "response time period", they could examine fluctuations within the same time period. Moreover, if the authors presented such trial-averaged EEG signals, this would give more convincing evidence that the experimental task has some measureable effect on the EEG signals. Finally, it would probably make the most sense to seek task related changes on an EEG channel near auditory cortex rather than channel Cz (a channel map should be provided in the manuscript for readers without expertise in EEG). As far as I can tell from the methods, the authors simply divide up the data into 10 s segments regardless of when the auditory stimulus is delivered. In my view, this lack of effort to identify the evoked response makes it somewhat ambiguous whether or not there is a significant difference in the variability of stimulus evoked response for young vs. old.”*

The procedure suggested by the reviewer is indeed appropriate to study the changes in task evoked responses from a conventional perspective where the averaging of time-series makes sense, or at least does not cause any harm. We are familiar with the procedure and it has been used by some of us with different types of analysis to study trial-averaged responses (cf., Müller et al., 2008, *BMC Neurosci*, and Müller et al., 2009, *Dev Sci*). However, in the present work, we capitalize on the functional meaning of EEG fluctuations and we study their changes with age in different functioning states (Rest, OddnC and OddC). The central point is not to understand directly how the task modifies cortical activity, but rather to understand whether groups’ differences in electrocortical variability change under more or less demanding experimental conditions. This informs us about changes in the dynamics of task-related (not stimulus-related) activity compared to resting state. Therefore, since we do not address the evoked responses, we have no principled reason to consider it more relevant to focus solely on fluctuations that are in the close neighborhood of the stimulus. In addition, it would be incongruent and methodologically inconsistent to apply multiscale variability metrics, which are our main outcome measures, on averaged, or even concatenated, stimulus-locked time-series. Indeed, meaningful information about time-structured fluctuations would be averaged out, and, time-series would become significantly shorter which precludes any multiscale analysis, or at the best, limits the analysis to very a few time-scales (e.g., McIntosh et al., 2013). In any case, there wouldn’t be a way to make resting-state and task-evoked activity strictly comparable (for instance, no comparison was made in that sense in the study of McIntosh et al., 2013). A justification acknowledging this issue was added to the revised manuscript (see EEG recordings and preprocessing, p.4). We also added a clarification regarding the segmentation, which for the task conditions, was done in a way ensuring a comparable number of stimuli between segments.

As for the suggestion of analyzing channels that are closer to the auditory cortex, we point out to the reviewer that all channels were analyzed and accounted for in the results’ description. This is justified by the fact that, in terms of changes in brain dynamics, we expected a global modification of EEG fluctuations with age, in both task and resting state, which was actually confirmed by our results.

The Cz electrode was only used as a visualization. Its choice was not random since it is generally less affected by muscle artifacts, and it is relevant to the studied task. Indeed, the oddball task has been shown to involve other regions in addition to those of the auditory cortex, to which the central electrode (Cz) appears to be quite representative (see Müller et al., 2008, 2009). These clarifications were added in the revised manuscript (see Results, p.10). We also added channel labels to the brain maps figure (see Fig. 7).

MINOR POINTS

**pg 1 lines 9-11**: “This sentence sounds like the age-related changes only occurred in time-scale dependent experimental conditions. Consider rewriting in two sentences for clarity”

The sentence was reformulated as follows: “In both task and rest conditions, our results mainly revealed that EEG signals presented systematic age-related changes that were time-scale dependent with regard to the structure of fluctuations (complexity) but not with regard to their magnitude”.

**pg 2 lines 4-13**: “*This seems like a 'pet theory' that is rather disconnected from the experimental data presented. It is better suited to the discussion section”*.

Actually, these opening lines introduce the theoretical positioning of the paper and motivate our interest in brain signal variability from the dynamical systems perspective. Although this paragraph does not deal with experimental data, we believe that it is a good entry point for a theoretically justified study. Nevertheless, following the reviewer’s comment, we have shortened it (see Introduction, p.2).

**pg 2 line 14**: “*Line 14 picks up with actual experiment facts that may be better suited to introduce the paper. Indeed, another theoretical perspective posits that there are in fact no stable states and that the system 'noise' is inherent to the dynamics as in chaotic or critical network dynamics. How does one distinguish between the situation A (stable states + noise) and situation B (inherently unstable dynamics). Perhaps this is a discussion point, but the intro should not be written as if situation A is the only possible underlying hypothesis.*”

We fully agree with the reviewer. Actually, in the initial manuscript, before discussing the role of noise (lines 14-22), the potential functional role of instability was presented by introducing the concept of ghost attractors (lines 8-13), corresponding to the reviewer’s situation B. Indeed, when at rest, the brain displays a complex though spatiotemporally structured dynamics, where brain states known as resting state networks (RSNs) are intermittently occupied. The RSNs have received particular attention, because several of them have been observed in task paradigms and have thus been hypothesized to have functional meaning (Deco et al., 2013). The mechanisms underlying the resting state dynamics remains a matter of debate. Within deterministic frameworks heteroclinic cycles have been proposed to generate sequential transitions from one unstable equilibrium point (in particular a saddle) to another, where each equilibrium point corresponds to a particular functional (cognitive, emotional, or behavioral) mode. Thus, robust, transient sequential dynamics can be generated that are characterized by asymptotic stability in a neighborhood of the state space resembling a hypertube (see Figure 1.3 of Krupa & Melbourne, 2008). Other deterministic approaches soften the requirement of unstable states and require attractive subspaces, which may contain attractors expressing functionally relevant dynamics (so-called Structured Flows on Manifolds, see Huys et al 2014). These approaches are subject to noise, but do not necessarily require the latter as a generative element.

We have now explained this briefly in the revised introduction and made sure to clarify that we are not speculating in favor of one possibility over the other (cf., introduction p.2).

**pg 5 lines 5-8**: It appears that your variable names are mixed up here. For example, if each time series has 2500 points sampled at 4 ms intervals, then for T=16 ms, the number of samples per window should be Nw=4 and the number of windows should be W=625.

We thank the reviewer for identifying this mistake. Now those lines have been changed to: “Thus, the number of windows W(s) ranged as W = 625…50, and the number of samples per window as Nw = 4…50, respectively.”

**pg 5 line 26**: Was the 'variogram' developed by the authors or has it been used in previous work? Please cite it if it was used previously or state that it is a new metric if that is the case.

The presentation of the variogram has now been modified in accordance with the reviewer’s comment (see Metrics, p.6) and we added a reference to the original method (Cressie, 1993), as well as to its (limited) use in neuroscience (Conte et al., 2009).

**pg 5 line 29**: This should be, "... decays according to a power-law with exponent lower or ...". Other types of decay can have well defined mean.

The reviewer is right; the sentence was not properly formulated and is now corrected.

**pg 5 line 33**: Some intuition for interpreting the variogram would be nice. For example, how does it behave for white noise, pink noise, periodic signals?

The reviewer can appreciate the results of applying the variogram analysis on several test signals in the figure below (Fig. A). However, since eNeuro does not allow the addition of appendices, we refrain from adding a figure with test signals to the manuscript, which is also in line with the previous comment on redundancy. Otherwise, since the variogram is not a new metric that we have introduced, one could demand to do the same with all measures, which would lengthen the Methods section even more. Nevertheless, we added more information to the description of the variogram analysis and some intuition on how to interpret the different measures in the Methods section. Figures 1 and 2 of the revised manuscript can now be used for the same purpose. We also discuss in the caption of Figure 2 the relationship between the power spectrum and the variogram.

**Fig. A. Variogram results with different test signals**: white noise (grey), brown noise (brown), pink noise (pink), Ornstein-Uhlenbeck process (green), 10 Hz non-linear oscillator with (red) and without noise (blue).

**pg 6 line 8-9**: Again, the W and Nw variables are switched.

We again thank the reviewer for this observation. The lines have been now corrected to: “Thus the number of windows W(s) ranged as W = 2500…50 and the number of samples per window as Nw = 1…50, respectively.”

**Figure 1**: “*Although the differences across groups (young vs. old) are clear, I have some concerns about interpreting the various measures that are presented in this figure. The power spectra clearly exhibit a peak around 10 Hz, which suggests that fluctuations at a time scale around 100 ms should be large. This seems to be at odds with the various other measures of fluctuations versus time scale. For example, there is no peak in SD around 100 ms. In fact, there is a decrease in ln(V) near 100 ms. These basic observations make it somewhat confusing to interpret the meaning of these measures. It would helpful if the authors could explain this.*

It would be also be helpful for understanding how the measures in the bottom three rows of Fig 1 are related if the horizontal axes were aligned and all plotted in the same way (i.e. all logarithmic or all linear).

It would also be nice to see some basic time series of the data. Jumping immediately into presenting the results of these complicated measures without ever seeing the data limits the understanding and interest of the reader.”

The confusion with regard to the reviewer’s attempt to link the results of the different measures stems from the fact that there is no direct and/or straightforward correspondence between frequencies of the power spectra, and time scales of the measures that required coarse graining (*MSE(n)*, *MLZ(n)* and *SD*), or detrending (*log(F)* of *DFA*). This was not clear in the initial submission. Following the reviewer’s comment, we now discuss this issue, along with the relationship between the power spectrum and the variogram (*log(V)*), in the context of Figures 1 and 2, as well as in the Methods section (p.4,6).

Regarding the linear against logarithmic scaling of plotting, we chose to use the standard way these metrics are presented in the literature, which relates to their interpretation, but sacrifices their direct comparability. Again, we are restricted by the lack of appendices that prevents us from providing figures with different scaling as well.

As requested, some EEG traces are now provided in Figure 1.
